# In Patients Treated by Selective Internal Radiotherapy, Cellular In Vitro Immune Function Is Predictive of Survival

**DOI:** 10.3390/cancers15164055

**Published:** 2023-08-11

**Authors:** Aglaia Domouchtsidou, Ferdinand Beckmann, Beate Marenbach, Stefan P. Mueller, Jan Best, Ken Herrmann, Peter A. Horn, Vahé Barsegian, Monika Lindemann

**Affiliations:** 1 Institute for Transfusion Medicine, University Hospital Essen, University of Duisburg-Essen, Virchowstraße 179, 45147 Essen, Germany; ldomouchtsidou@gmail.com (A.D.); fsgbeckmann@googlemail.com (F.B.); beate.marenbach@uk-essen.de (B.M.); peter.horn@uk-essen.de (P.A.H.); 2 Department of Microbiology, General Anticancer Oncological Hospital “Agios Savvas”, 115 22 Athens, Greece; 3 Department of Nuclear Medicine, University Hospital Essen, University of Duisburg-Essen, 45147 Essen, Germany; stefanp.mueller@t-online.de (S.P.M.); ken.herrmann@uk-essen.de (K.H.); vahe.barsegian@helios-kliniken.de (V.B.); 4 Department of Gastroenterology and Hepatology, University Hospital Essen, University of Duisburg-Essen, 45147 Essen, Germany; jan.best@kk-bochum.de; 5 Department of Internal Medicine, University Hospital Knappschaftskrankenhaus Bochum, Ruhr University Bochum, 44892 Bochum, Germany; 6 Institute of Nuclear Medicine, Helios Kliniken, 19049 Schwerin, Germany

**Keywords:** selective internal radiotherapy, lymphocyte proliferation, ELISpot, interferon-gamma, interleukin-2, patient survival, immune checkpoint molecule

## Abstract

**Simple Summary:**

To treat malignant liver tumors, the liver can be irradiated locally with the injection of radioactive microparticles, by a therapy called selective internal radiotherapy. We could previously show that this treatment is correlated with an impaired immune function of white blood cells. As the liver is a well-perfused organ, we assume that during their passage through the liver, white blood cells become irradiated, which leads to this functional impairment. In the current study, we observed in 25 patients treated with selective internal radiotherapy that a higher degree of immunosuppression was predictive of a 2-to-3.5-times shorter survival. We assessed the immune function radioactively by a cell proliferation assay, after stimulation with four microbial antigens.

**Abstract:**

In patients with liver malignancies, the cellular immune function was impaired in vitro after selective internal radiotherapy (SIRT). Because immunosuppression varied substantially, in the current study, we investigated in 25 SIRT patients followed up for ten years whether the lymphocyte function was correlated with survival. Peripheral blood mononuclear cells were stimulated with four microbial antigens (tuberculin, tetanus toxoid, *Candida albicans* and CMV) before therapy and at four time points thereafter, and lymphocyte proliferation was determined by H3-thymidine uptake. The median sum of the responses to these four antigens decreased from 39,464 counts per minute (CPM) increment (range 1080–204,512) before therapy to a minimum of 700 CPM increment on day 7 after therapy (0–93,187, *p* < 0.0001). At all five time points, the median survival in patients with weaker responses was 2- to 3.5-fold shorter (*p* < 0.05). On day 7, the median survival in patients with responses below and above the cutoff of a 2 CPM increment was 185 and 523 days, respectively (χ^2^ = 9.4, *p* = 0.002). In conclusion, lymphocyte function could be a new predictor of treatment outcome after SIRT.

## 1. Introduction

Liver cancer is the third leading cause of cancer-related death worldwide, and hepatocellular carcinoma (HCC) accounts for the majority of cases [1]. The treatment of unresectable HCC remains a challenge, with new combination regimens emerging and gaining approval in the clinical practice [2]. Selective internal radiotherapy (SIRT), a third-line therapy usually applied to patients of an advanced age, is a radioembolization technique consisting of Y^90^-loaded glass or resin microspheres which deliver radiation directly into the tumor via the hepatic artery [3]. There are compelling data that SIRT is a safe locoregional treatment for patients with intermediate and advanced HCC, exhibiting good tumor response and low toxicity [4].

Evidence suggests that local therapies may be successfully combined with cancer immunotherapies targeting immune checkpoint receptors [5,6]. Immune checkpoint blockade with monoclonal antibodies targeting the programmed death-ligand 1 (PD-L1)/programmed cell death 1 (PD-1) pathway or cytotoxic T lymphocyte-associated antigen 4 (CTLA-4) prolonged patient survival, considering many cancer types [7]. Especially, for the treatment of unresectable HCC, immune checkpoint inhibitors have shown promising results in terms of overall survival or progression-free survival [8,9,10]. Additionally, they can be used as an alternative when other systemic cancer treatments, such as sorafenib, are contraindicated [11].

Although it is now generally accepted that tumor–immune cell interactions are highly relevant for patient survival, data on the immune function after local treatment with radionuclides are scarce [12,13,14,15]. They indicate that depending on the radionuclide and its biodistribution in the body, the immune function could be influenced differently. Lymphocyte proliferation and interferon-gamma production were increased after radioiodine treatment of patients with thyroid carcinoma [13] but decreased after yttrium-90 DOTATOC therapy in patients with neuroendocrine tumors [12] or SIRT with yttrium-90 in patients with non-resectable hepatic malignancies [15].

As T cells are involved not only in antimicrobial T cell responses but also in tumor control, we hypothesized that those patients with worse T cell immunity should survive for a shorter time. In the current project, we analyzed if cellular in vitro function was predictive of patient survival after SIRT with glass microspheres (Therasphere™, Boston Scientific, Marlborough, MA, USA). The patients were followed up for approximately 10 years. Prediction of survival was not analyzed in our previous study, which only described changes of immune function within 28 days after SIRT [15]. To address this question, the peripheral blood mononuclear cells (PBMC) of 25 patients with non-resectable hepatic malignancies were stimulated prior to therapy and at four time points after therapy with four microbial antigens, and their proliferation was determined by H3-thymidine uptake.

## 2. Materials and Methods

### 2.1. Volunteers

This study included 25 patients (median age 73 years (interquartile range (IQR) 70–75), 5 females, 20 males) with unresectable HCC who were treated by SIRT at the University Hospital Essen between January 2013 and April 2014 and who were followed up until March 2023, i.e., for approximately 10 years (Table 1). Further details on this cohort have been published previously [15]. In the current study, the data were re-analyzed with respect to prediction of survival. The analysis included blood samples drawn prior to radiotherapy (day 0), directly after radiotherapy (1 h) and at day 2, 7 and 28 after therapy.

The study received institutional review board approval by the ethics committee of the Medical Faculty, University Hospital Essen, Germany (approval number 09-3991), and was carried out in accordance with the 1964 Helsinki Declaration.

### 2.2. Lymphocyte Transformation Test

The measurement of lymphocyte proliferation in the presence of the four microbial antigens tuberculin, tetanus toxoid, *Candida albicans* and CMV was described previously in detail [15]. In brief, after stimulation with the microbial antigens (a sextuplicate of cultures for each of them), the uptake of H3-thymidine by PBMC was determined in six-day cell cultures, and the proliferative responses (counts per minute, CPM) of 50,000 PBMC are presented as CPM increment, i.e., the antigen-specific response minus a value corresponding to the negative control. When calculating antigen-specific reactions and negative controls, the second highest value of six results was considered in each case to exclude possible outliers. The four CPM increment values were then summed up (cumulative CPM increment). Of note, cumulative antigen responses were also assessed by another group [16], and our group could show in immunocompromised patients that the cumulative antigen responses were more informative than the responses to individual antigens [17].

### 2.3. Statistical Analysis

Statistical analysis was performed with GraphPad Prism 8.4.2.679 (San Diego, CA, USA) or IBM SPSS Statistics version 25 (Armonk, New York, NY, USA). We compared the cellular in vitro responses at various time points by 1-way ANOVA. We used receiver operating characteristic (ROC) curve analysis und compared the groups by the Log Rank (Mantel–Cox) test, where survival was included as a dichotomous variable (above or below the median). Two-sided *p* values < 0.05 were considered significant.

## 3. Results

### 3.1. Lymphocyte Proliferation in Patients Prior to and Post SIRT

**Figure 1:**
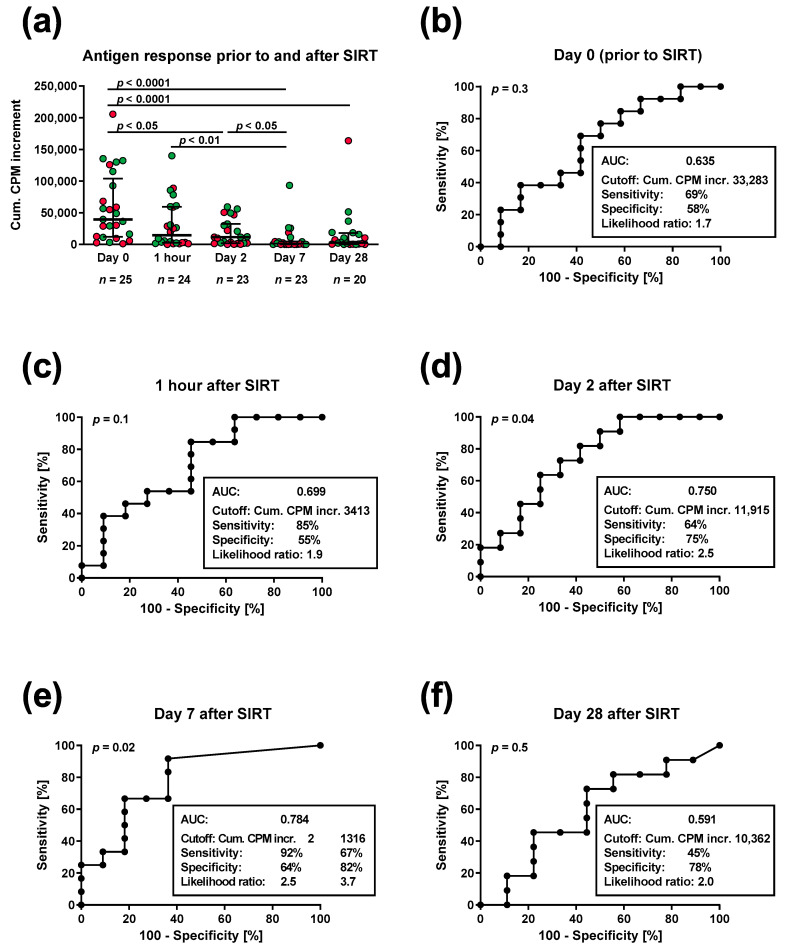
**Cellular response towards four microbial antigens in 25 patients treated by selective internal radiotherapy (SIRT).** Panel (**a**) shows individual data prior to SIRT and one hour and on day 2, 7 and 28 thereafter, together with median and interquartile range. Red dots indicate values for patients with a short survival (<median of 369 days), and green dots those for patients with a long survival (≥median). Data were compared by 1-way ANOVA, and the comparison of data from all test points yielded a *p* value of <0.0001. *p* values in panel (**a**) indicate the significance of pairwise comparisons, corrected for multiple comparisons. Panel (**b**–**f**) indicate the results of a receiver operating characteristic (ROC) curve analysis of the cellular response towards four microbial antigens and of survival (<median vs. ≥median). Each of these panels provides information on area under the curve (AUC), cutoff, sensitivity, specificity and likelihood ratio. On day 7 (panel (**e**)), we considered two different cutoffs for the cumulative (cum.) antigen response, as we observed two maxima for the likelihood ratio. CPM incr., counts-per-minute increment, i.e., antigen-specific response minus the negative control.

The PBMC of 25 patients with non-resectable hepatic malignancies were stimulated prior to SIRT and one hour and on day 2, 7 and 28 after SIRT with four microbial antigens (tuberculin, tetanus toxoid, *Candida albicans* and CMV), and their proliferation was determined by H3-thymidine uptake. The cumulative antigen response towards these four microbial antigens) decreased from a median CPM increment value of 39,464 (range 1080–204,512) before therapy to 14,474 (43–139,794) one hour after therapy, 11,479 (0–59,206) on day 2, 700 (0–93,178) on day 7 and 2750 (0–163,738) on day 28 (*p* < 0.0001 for all test dates and for day 0 vs. day 7 or day 0 vs. day 28, respectively) (Figure 1a). Thus, the responses reached a minimum on day 7 after therapy. Of note, particularly on day 7 after SIRT, the patients with a short survival (<median of 369 days after SIRT) had a lower cumulative antigen response as indicated by red dots, which was further examined by ROC curve analysis.

### 3.2. Lymphocyte Proliferation and Patient Survival after SIRT

The patients were divided into two groups, with short and long survival, separated by the median. All patients died within a follow-up period of ten years (day 35–2073). ROC curve analysis indicated that the cumulative antigen responses on day 2 and day 7 after therapy were significantly predictive of patient survival (*p* = 0.04 and *p* = 0.02, respectively) and that the largest area under the curve (AUC) was obtained on day 7 (Figure 1b–f). Prior to SIRT, the AUC was 0.645, one hour after SIRT, it was 0.699, on day 2 after SIRT, 0.750, on day 7 after SIRT, 0.784 and on day 28 after SIRT, 0.591. On day 7 after SIRT, the ROC curve analysis yielded two maxima for the likelihood ratio, when setting the cutoff for the cumulative CPM increment at 2 (sensitivity 92%, specificity 64%) or at 1316 (sensitivity 67%, specificity 82%) (Figure 1e). Of note, survival was considered as a dichotomous variable (short vs. long) in this ROC curve analysis.

**Figure 2:**
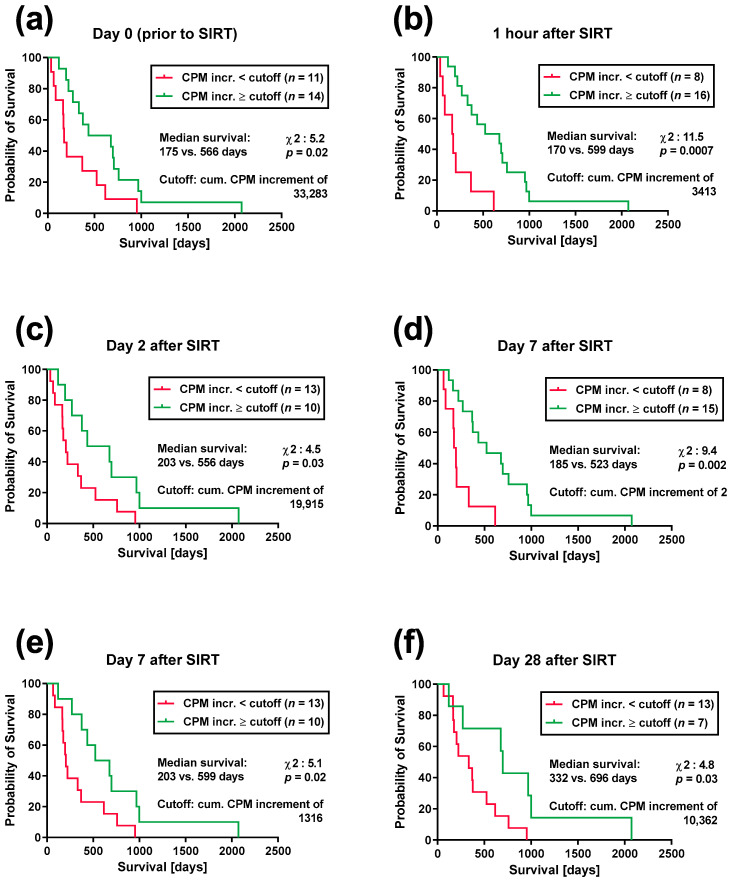
**Impact of the cumulative antigen response on the survival of 25 patients treated by selective internal radiotherapy (SIRT).** Panel (**a**) shows the survival curves with respect to the cumulative (cum.) antigen response (towards four microbial antigens) prior to SIRT, and panel (**b**–**f**) show curves after SIRT. Please note that the analysis on day 7 after SIRT was performed with two different cutoff values, as explained in the legend to Figure 1**e**. Survival curves were compared by the Log Rank (Mantel–Cox) test. CPM increment, counts-per-minute increment, i.e., antigen-specific response minus the negative control.

The comparison of the survival curves by the Log Rank (Mantel–Cox) test confirmed the data of the ROC curve analysis on day 2 and on day 7 after SIRT and furthermore yielded significant results for the remaining time points (*p* values between 0.03 and 0.0007) (Figure 2). Thus, the patients with cumulative antigen responses below a certain cutoff—that was defined by the ROC curve analysis—had a 2- to 3.5-times lower median survival than those exceeding the respective cutoff. Irrespective of the time point, the survival curves were similar. Prior to therapy, eleven SIRT patients with a cumulative antigen response of less than 33,283 CPM increment had a median survival of 175 days, whereas those 14 SIRT patients with a response exceeding the cutoff had a median survival of 566 days, i.e., the median survival of the latter group was 3.2-fold longer (χ^2^ = 5.2, *p* = 0.02) (Figure 2a). One hour after SIRT, the patients with antigen responses below and those with antigen responses above the cutoff had a median survival of 170 and 599 days, respectively (3.5-fold difference, χ^2^ = 11.5, *p* = 0.0007) (Figure 2b). On day 2 after SIRT, the difference in median survival was 2.7-fold (Figure 2c), on day 7 it was—depending on the cutoff—2.8-fold (Figure 2d) or 3.0-fold (Figure 2e), and on day 28 it was 2.1-fold (Figure 2f). On day 7, we set the cutoff either at the cumulative CPM increment value of 2 (Figure 2d) and obtained a median survival of 185 vs. 523 days (χ^2^ = 9.4, *p* = 0.002) or at a value of 1316 (Figure 2e), obtaining a median survival of 203 vs. 599 days (χ^2^ = 5.1, *p* = 0.02) for patients with antigen responses below and above the cutoff, respectively.

## 4. Discussion

We previously showed that patients treated with SIRT for unresectable hepatic malignancies exhibit a strong immunosuppression after therapy. In the present study, it was revealed for the first time that the severity of the immunosuppression could predict the survival rate, i.e., patients whose immune cells were incapable to react and proliferate in vitro after contact with the antigens survived for a significantly shorter time compared to those with a reduced but yet sustained immune response. Overall, the median survival of the patients with higher cumulative antigen scores was 2- to 3.5-fold longer, and this was evident at all time points, i.e., prior to therapy, 1 h, 2 days, 7 days and 28 days after therapy.

One hour after the injection of the radioactive microspheres, the median lymphocyte proliferation already decreased to 37% of the baseline value (Figure 1a). The administered radiation was at its peak one hour after therapy and continuously decreased within the first week. Nevertheless, since the liver is a well-perfused organ and the half-life of ^90^Y is approximately 2.6 days (64.1 h), the lymphocytes are likely irradiated many times during their passage through the liver. In a previous study, we showed that the accumulation of DNA double-strand breaks increased within the first week and that the increase in this form of DNA damage was inversely correlated with lymphocyte proliferation, leading to a minimum of cellular immune responses on day 7 [18]. Most likely, the function of lymphocytes was hindered from the ongoing effort to repair DNA damages [19].

Evidence from other studies shows that not only the state of the whole immune system, but also the immunological tumor microenvironment can predict the overall survival [20,21,22]. A study by Tu et al. analyzed the tumor-infiltrating lymphocytes (TILs) in HCC and examined the role of TILs in predicting patient survival. They found that the intratumoral tissue of HCC was enriched with regulatory T cells (Tregs), compared to the peritumoral tissue, where CD3+, CD4+ and CD8+ T cells, NK cells and macrophages were abundant. These findings indicate that the tumor induces a rather immunosuppressive state. Most importantly, a higher percentage of tumor-infiltrating Tregs was an unfavorable prognostic factor for survival in HCC patients [23].

The role of the immune system in predicting the survival outcome and the consequences of the immunosuppressive state on promoting cancer cell proliferation have been demonstrated more conclusively after liver transplantation. Many studies corroborate the hypothesis that tumor recurrence is more likely to occur when higher doses of immunosuppressive agents, such as calcineurin inhibitors (CNIs), are administered [24,25,26]. It was proposed that a preventative measure to lower the risk of HCC recurrence would be to reduce the use of CNIs within the first month from liver transplantation [27]. Moreover, previous studies advocate that the reduction in the immunosuppressive regimen in liver recipients, upon a diagnosis of HCC recurrence or de novo lung cancer, will lead to improved survival [28,29]. Thus, the International Liver Transplantation Society strongly recommends a unique approach for each HCC patient when applying immunosuppression in the posttransplant phase, with focuses on minimizing the dosage of CNIs [30].

The main causes which lead to primary liver cancer are chronic hepatitis B and C, nonalcoholic fatty liver disease, aflatoxin B1 exposure and alcohol abuse [31]. Regardless of whether liver cancer is due to persistent viral hepatitis or metabolic disease of the liver, the forerunner is always the formation of a highly tumorigenic milieu based on non-resolving chronic inflammation [32,33]. In this environment, immunosuppressive phenomena enable cancer cells to evade the attack by the immune system and therefore justify immunomodulatory interventions. The use of immune checkpoint inhibitors against PD-1/PD-L1 and CTLA-4 for HCC is being evaluated in clinical trials, and promising results are on their way [34]. Nevertheless, data suggest that combination regimens with locoregional treatments will be needed to increase the effectiveness of immune checkpoint inhibitors [35,36].

In our study, patients with nonresectable HCC treated with SIRT showed impaired immune functions in vitro, i.e., reduced T cell activation and proliferation. Moreover, we could show that these weak immune reactions in vitro correlated with higher radiation-induced double-strand DNA lesions [18]. In an attempt to elucidate other reasons for this prompt and severe immunosuppression after radiotherapy, as well as to find possible ways to enhance the immune reaction, we investigated the involvement of the PD-1/PD-L1 pathway. We assumed that the blockage of the PD-1/PD-L1 pathway in vitro could lead to better immune responses. However, our preliminary results failed to confirm this hypothesis (Figure S1). Lymphocyte proliferation did not increase after adding monoclonal antibodies against PD-1 and PD-L1, indicating that the PD-1/PD-L1 pathway may not be involved in the observed immunosuppression. Checkpoint inhibitors have revolutionized cancer treatment. Nevertheless, many patients display primary resistance to immunotherapy [37]. In lack of good biomarkers predicting poor responders [38,39] and due to the small number of patients in this subgroup, we cannot exclude that poor response to PD-1/PD-L1 blockage could also be a reason for our results.

To assess the correlation between administered radiation activity and cellular immune responses, we performed Spearman correlation analyses. We found that the sum of antigen responses on day 7 correlated significantly with the administered radiation activity (GBq) (*r* = −0.57, *p* = 0.005). A similar trend was observed on day 2 (*r* = −0.36, *p* = 0.09) and day 28 (*r* = −0.37, *p* = 0.10). Thus, the administration of a higher radiation activity led to decreased in vitro immune responses. A more extensive analysis of the correlation between various clinical parameters and lymphocyte responses towards twelve different antigens and four mitogens was described in a previous publication [15]. It was revealed that apart from a higher radiation activity, the presence of liver cirrhosis, chronic kidney disease, diabetes mellitus and metastases was unfavorable for the immune function, while a better Eastern Cooperative Oncology Group (ECOG) performance status led to better in vitro immune reactions.

In order to measure the proliferative ability of peripheral white blood cells after radioembolization, we used the lymphocyte transformation test (LTT), which requires the use of radioactive thymidine in up-to-six-day cell cultures. Searching for an immunoassay that is sensitive and more suitable for clinical routine use in order to predict patient survival, we established an ELISpot assay detecting the production of the pro-inflammatory cytokine IL-2. During an immune response, IL-2 is secreted in large amounts by activated antigen-specific T cells [40] and can be used as a marker of immunocompetence. Our current experiments indicated that one day after SIRT, the cellular responses tended to be similarly reduced, both in the LTT and in the IL-2 ELISpot assay (Figure S2). We believe that IL-2 ELISpot has the potential to diagnose alterations in the immune system after SIRT. A larger cohort of patients with a longer follow-up is required in order to explore whether the IL-2 ELISpot results would correlate with the survival rate.

## 5. Conclusions

We showed that in 25 patients treated by SIRT, severe immunosuppression was significantly predictive of shorter survival (*p* = 0.002). Our results, together with other current studies trying to elucidate the highly complex tumor microenvironment in the liver, may offer new insights into the potential effectiveness of combination therapies involving radioembolization in patients with liver cancer or liver metastases.

## Figures and Tables

**Table 1:** Characteristics of the 25 patients treated by selective internal radiotherapy.

Parameter		Absolute Number or Median (Range)
Age (years)		73 (33–84)
Sex	Female	5
	Male	20
Body mass index (kg/m^2^)		26.6 (20.1–38.3)
Comorbidities	Kidney insufficiency	5
	Diabetes mellitus	13
	Hypertension	22
	Coronary heart disease	11
ECOG performance status	0	14
	1	9
	2	2
Kidney function	Serum creatinine (mg/dL) ^b^	1.0 (0.8–2.2)
	GFR (mL/min/1.73 m^2^) ^b^	69 (31–113)
	Albumin (g/dL) ^b^	4.1 (3.4–4.9)
Liver function	Aspartate aminotransferase (U/L) ^b^	52 (28–227)
	Alanine aminotransferase (U/L) ^b^	36 (17–208)
	Alkaline phosphatase (U/L) ^b^	148 (64–775)
	Bilirubin, total (mg/dL) ^a^	0.8 (0.4–1.8)
	Gamma-glutamyl transferase (U/L) ^b^	184 (20–1467)
	Quick (%)	97 (42–120)
Median administered activity (GBq)		2.9 (1.0–6.4)
Therapy volume (Gy)		119 (110–282)
Dose to the lungs (Gy) ^a^		6.5 (1.0–21.4)
Location of primary tumor	Liver	24
	CUP	1
Metastases	Without	10
	Lungs	4
	Adrenal gland	2
	Bones	2
	Pancreas	1
	Stomach	1
	Other	5

ECOG, Eastern Cooperative Oncology Group; GFR, glomerular filtration rate; CUP, cancer of unknown primary. ^a^ One unknown value, ^b^ two unknown values.

## Data Availability

The data presented in this study are available on request from the corresponding author. The data are not publicly available due to privacy restrictions.

## Supplementary Materials

The following supporting information can be downloaded at: https://www.mdpi.com/article/10.3390/cancers15164055/s1, Figure S1: Impact of the immune checkpoint molecules PD-1/PDL-1 on lymphocyte proliferation; Figure S2: Results of the IL-2 ELISpot assay prior to and after selective internal radiotherapy.
