# Supplementary material for: In Patients Treated by Selective Internal Radiotherapy, Cellular In Vitro Immune Function Is Predictive of Survival
Source: Cancers (Basel). 2023 Aug 11;15(16):4055. doi: 10.3390/cancers15164055 (PMC10452121; doi:10.3390/cancers15164055)
Supplement: Supplementary file 1 [file cancers-15-04055-s001.zip › cancers-2513359-supplementary.pdf]

Supplementary Materials

Impact of immune checkpoint molecules PD-1/PDL-1 on lymphocyte proliferation

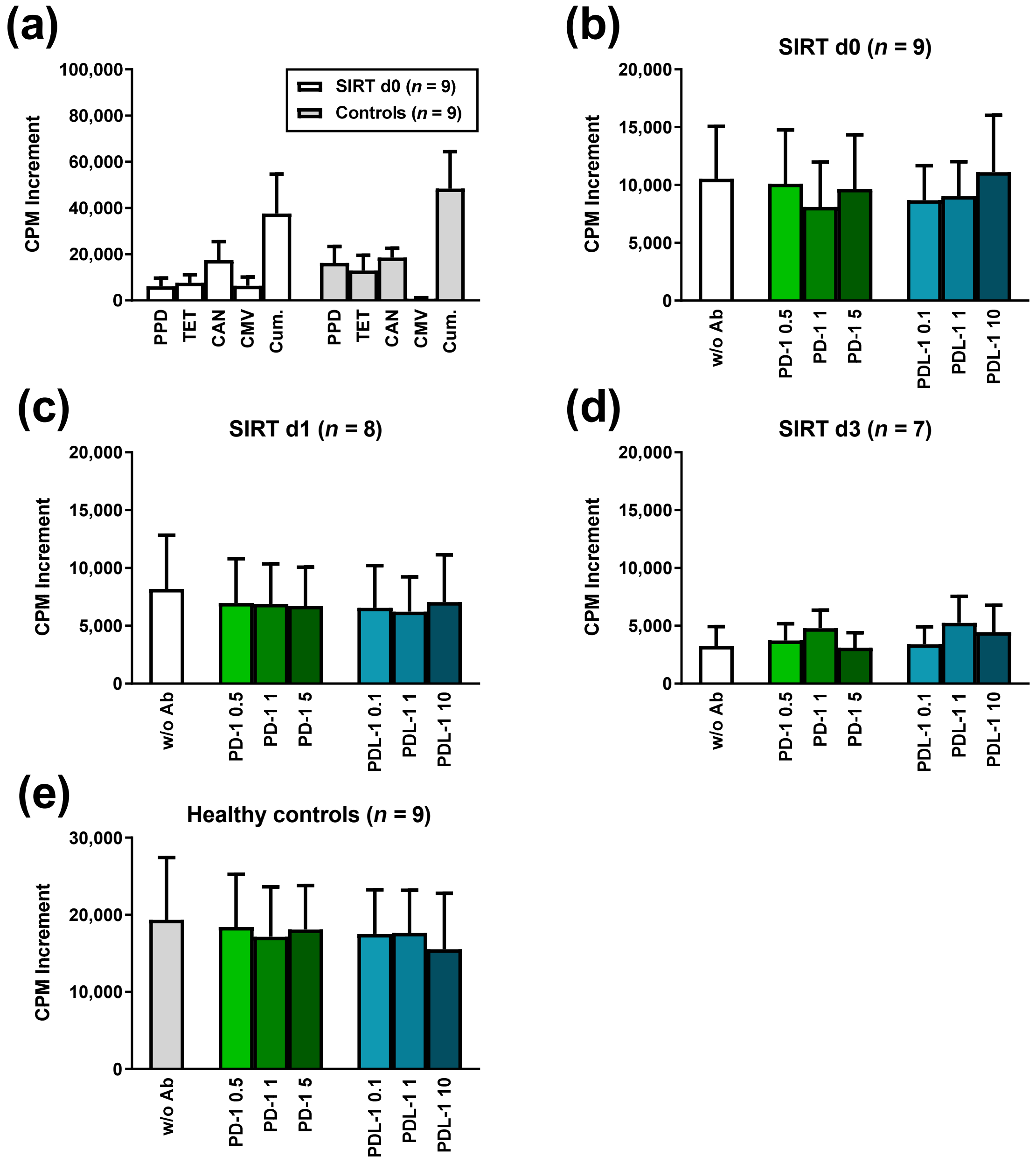

Figure S1. Cellular responses towards microbial antigens in patients prior to and after selective internal radiotherapy (SIRT) and in healthy volunteers without and with antibodies against PD-1/PDL-1. To assess the impact of immune checkpoint molecules PD-1/PDL-1 nine additional SIRT patients (median age 69 years (IQR 57-72), 2 female, 7 male) and nine healthy volunteers (median age 44 years (IQR 26-60), 2 female, 7 male) were analyzed. The patients were tested by lymphocyte transformation test prior to SIRT and on day 1 and day 3 thereafter. To find out if this cohort showed a similar response as the 25 SIRT patients presented in detail in the current paper, proliferative responses to four microbial antigens were determined in parallel. Panel (**a**) shows proliferative responses towards the antigens tuberculin (purified protein derivate, PPD), tetanus toxoid (TET), *Candida albicans* (CAN) and CMV and the sum of the four responses (cumulative response, Cum.). As compared to patients prior to SIRT (day 0), the matched healthy controls had overall slightly higher ($p > 0.1$) proliferative responses (Mann-Whitney test). The strength of antigen responses was in a similar range as in the cohort of 25 SIRT patients. Panel (**b**) to (**e**) indicate results of blocking experiments with monoclonal antibodies against the immune checkpoint molecules PD-1/PDL-1 at concentrations of 0.1-10 µg/mL (both Thermo Fisher Scientific, San Diego, USA), which were added to cell cultures stimulated with PPD (75 µg/mL). Panel (**b**) indicates results of patients prior to SIRT (day 0), panel (**c**) at day 1 after SIRT and panel (**d**) at day 3 after SIRT. For comparison, data on healthy controls are presented as panel (**e**). All data were given as mean and standard error of the mean (SEM). T cell responses with and without antibodies against PD-1/PDL-1 (**b**-**e**) were compared by 1-way ANOVA (corrected for multiple comparisons), which did not yield significant differences. CPM increment-counts per minute increment, i.e. antigen specific response minus negative control (unstimulated).

Results of the IL-2 ELISpot assay prior to and after selective internal radiotherapy

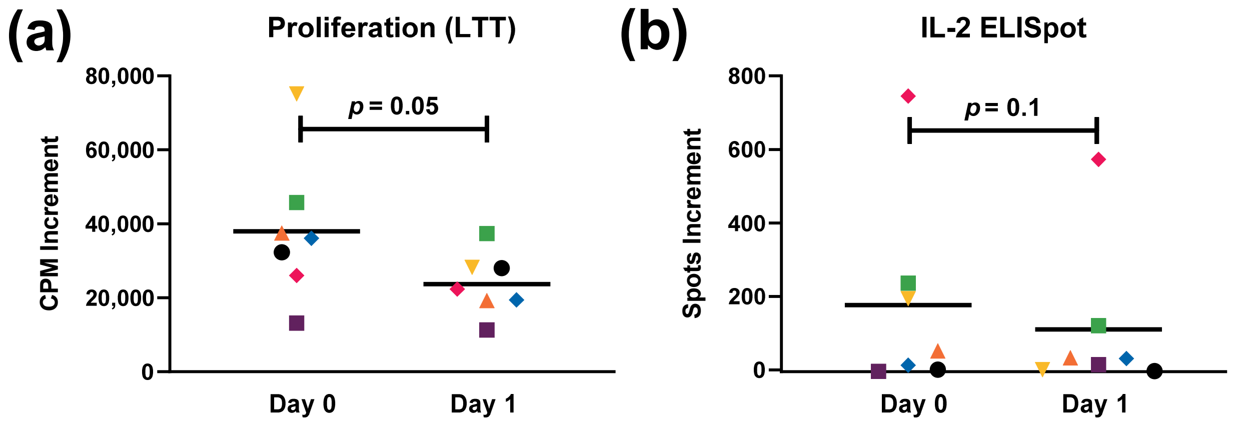

Figure S2. Cellular responses towards pokeweed mitogen in patients treated by selective internal radiotherapy (SIRT). In seven patients (median age 65 years (IQR 59-75), 2 female, five male) prior to selective internal radiotherapy (SIRT) and at day 1 (3-5 hours after SIRT), proliferative and ELISpot responses towards pokeweed mitogen (PWM) were determined in parallel. Proliferative responses were assessed by lymphocyte transformation test – as described in the *Methods* section – in four-day cultures of 50,000 PBMC. ELISpot experiments were performed with stripes containing PVDF membranes (MultiScreen HTS, Fisher Scientific, Schwerte, Germany). After activation with 35% ethanol the plates were coated for 3 h with 60 µL of monoclonal antibodies against IL-2 (10 µg/mL of clone MT2A91/2C95, Mabtech, Nacka, Sweden). Thereafter, ELISpot plates were washed and blocked and then duplicates of 125,000 PBMC were grown in the presence or absence of the PWM. After 19 h incubation at 37°C, the ELISpot plates were washed and captured IL-2 was detected by incubation for 1 h with 50 µl of the biotinylated monoclonal antibody against IL-2 (5 µg/mL of clone MT8G10 biotinylated, Mabtech), diluted with phosphate-buffered saline plus 0.5% bovine serum album. After further washing, 50 µL of nitro blue tetrazolium/5-bromo-4-chloro-3-indolyl-phosphate was added and purple spots appeared within 7 min. Spot numbers were analyzed by an ELISpot reader (AID Fluorospot, Autoimmun Diagnostika GmbH, Strassberg, Germany). Panel (**a**) shows individual data and the mean values of proliferative responses prior to SIRT (day 0) and at day 1 after SIRT, panel (**b**) the results of the IL-2 ELISpot. Data were compared by Wilcoxon matched pairs test. CPM-counts per minute, Increment-PWM stimulated response minus negative control (unstimulated).
